# Fluctuation-Mediated Spin–Orbit Torque Enhancement in the Noncollinear Antiferromagnet Mn_3_Ni_0.35_Cu_0.65_N

**DOI:** 10.1021/acs.nanolett.4c05423

**Published:** 2025-05-13

**Authors:** Arnab Bose, Tom G. Saunderson, Aga Shahee, Lichuan Zhang, Tetsuya Hajiri, Adithya Rajan, Durgesh Kumar, Dongwook Go, Hidefumi Asano, Udo Schwingenschlögl, Aurelien Manchon, Yuriy Mokrousov, Mathias Kläui

**Affiliations:** † Institute of Physics, Johannes Gutenberg-University Mainz, Staudingerweg 7, Mainz 55128, Germany; ‡ Department of Electrical Engineering, Indian Institute of Technology, Kanpur 201086, UP, India; § Peter Grünberg Institut and Institute for Advanced Simulation, Forschungszentrum Jülich and JARA, Jülich 52425, Germany; ∥ School of Physics and Electronic Engineering, 12676 Jiangsu University, Zhenjiang 212013, China; ⊥ Department of Materials Physics, 12965 Nagoya University, Nagoya 464-8603, Japan; # Physical Science and Engineering Division, 127355 King Abdullah University of Science and Technology, Thuwal 23955-6900, Saudi Arabia; ∇ 128791 Aix-Marseille Université, CNRS, CINaM, Marseille 13009 France; ○ Centre for Quantum Spintronics, Norwegian University of Science and Technology, 7491 Trondheim, Norway

**Keywords:** spin and orbital Hall effect, noncollinear antiferromagnet, spin−orbit torques, spin fluctuation

## Abstract

We report strong spin–orbit torques (SOTs) generated by noncollinear antiferromagnets Mn_3_Ni_0.35_Cu_0.65_N, over a wide temperature range. The SOT efficiency peaks up to 0.3 at the Néel temperature (*T*_*N*_), substantially higher than that of commonly studied nonmagnets, such as Pt. The sign and magnitude of the SOTs measured in our experiments are corroborated by density functional theory, confirming the dominance of the orbital Hall effect over the spin Hall effect in the nonmagnetic phase above *T*_*N*_. In contrast, the strong temperature-dependent SOTs observed around and below *T*_*N*_ can be explained by recently developed mechanisms involving chirality-induced and extrinsic scattering-driven spin and orbital currents, considering the effect of spin fluctuations at finite temperatures. Our work not only reports a large magnitude of SOT but also sheds light on a new possible origin where orbital currents can be harnessed by leveraging the chirality of noncollinear antiferromagnets, which holds promise for magnetic memory applications.

## Graphical abstract

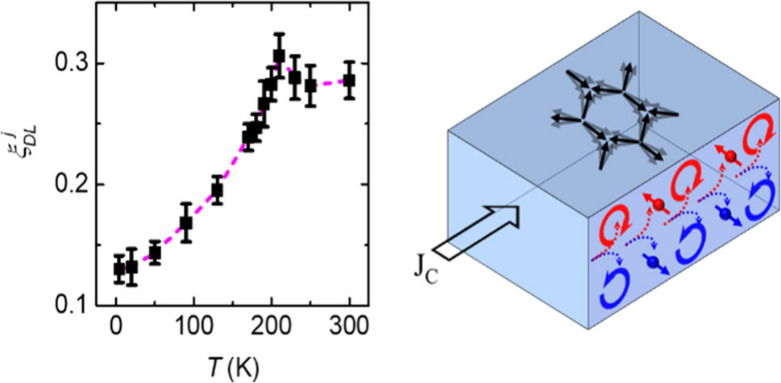

Spin–orbit torques (SOTs) currently offer a highly efficient mechanism for the current-induced switching of nanomagnets as required for the implementation of next-generation spintronics devices such as nonvolatile magnetic random-access memories (MRAM).^1,2^ Typically, strong SOTs are generated by applying an in-plane electric current in the heavy metal (HM)/ferromagnet (FM) bilayers, where the HM generates a transverse spin current due to the spin-Hall effect (SHE).^3^ Recently, spintronics research has shifted its focus toward generating orbital Hall currents^4^ in nonmagnets (NMs). This shift is primarily due to the advantage that orbital Hall currents are not limited by spin–orbit coupling (SOC), thereby broadening the range of materials that can serve as promising candidates for MRAMs.^5^ On the other hand, noncollinear antiferromagnets (NC-AFMs) represent a new class of magnetic materials that exhibit various exotic effects,^6^ including chirality-induced anomalous Hall effect^7–11^ without requiring net magnetization and unconventional spin currents,^12–19^ although studies on orbital Hall effects (OHE) in these materials are still lacking. Previously, a chirality-driven novel spin current has been predicted to exhibit a strong temperature dependence due to spin fluctuations in NC-AFM systems,^14^ yet this phenomenon has not been experimentally reported. Some recent works also suggest a significant effect on the orbital properties of conducting electrons in magnets induced by spin fluctuations.^20,21^ Therefore, exploring novel spin and orbital current generation over a wide temperature range in NC-AFMs is of fundamental interest.

The orbital current is a fundamental quantity primarily generated from the orbital character of the bandstructure, providing an intrinsic robust source^4^ that can manifest as a spin current in the presence of spin–orbit coupling (SOC).^22–25^ A nontrivial temperature dependence of the spin current has been theoretically predicted in magnetic materials, including ferromagnets^26^ and collinear and noncollinear antiferromagnets,^14,27^ due to spin fluctuations, which contribute to extrinsic scattering mechanisms such as skew scattering and side jump.^3,14,26,28^ Such fluctuation-mediated spin-current generation has been experimentally reported in ferromagnets^29–31^ and collinear AFMs recently.^27,32^ Magnon transport through a collinear insulating AFM spacer layer has been exploited to probe magnetic phase transition.^33–35^ These observations raise an intriguing question of whether spin fluctuations in chiral NC-AFMs can be utilized to produce large SOTs for practical applications. In this work, we address this key question while exploring the role of orbital current influenced by the chirality of NC-AFM. We have studied SOT produced by NC-AFM, Mn_3_Ni_0.35_Cu_0.65_N (MNCN), as its Néel temperature (*T*_*N*_) is around 210 K, providing full access to quantify SOT from cryogenic temperatures to room temperature.

We prepare epitaxial MNCN films of a thickness of 15 nm by the reactive magnetron sputtering technique on a (111) oriented single-crystal MgO substrate (Figure 1a). More details of the thin-film growth and material characterization can be found in the Supporting Information and previous works.^10,36^ After the MNCN growth, Cu (1.5 nm) /Ni_81_Fe_21_ (Py) (4–6 nm)/Cu (2 nm)/AlO_*x*_ (2 nm) is deposited without breaking the vacuum. We use a 1.5 nm Cu spacer between MNCN and Py to magnetically decouple these layers, while the 2 nm thick top Cu layer is used to reduce the overall Oersted field acting on the magnet by the current shunting effects through both the top and bottom layers. The decoupling is necessary as the effect on the Py could not be measured for exchange-coupled antiferromagnet and ferromagnetic layers. So only by decoupling, the Cu layer enables a clear detection of damping-like torque (DLT) acting on the Py in our experiment. Py is used as a magnet as it shows nominal in-plane anisotropy compared to other elemental magnets, such as Co and Ni, when grown on epitaxial materials such as MNCN. High Ni concentrations further enable us to probe the contribution of the orbital current by leveraging its spin–orbit coupling. In MNCN, the Kagome plane resides in the (111) sample plane (more details in the Supporting Information) where Hall-bars (50 × 20 μm^2^) are fabricated using standard optical lithography, etching, and lift-off techniques.

**Figure 1:**
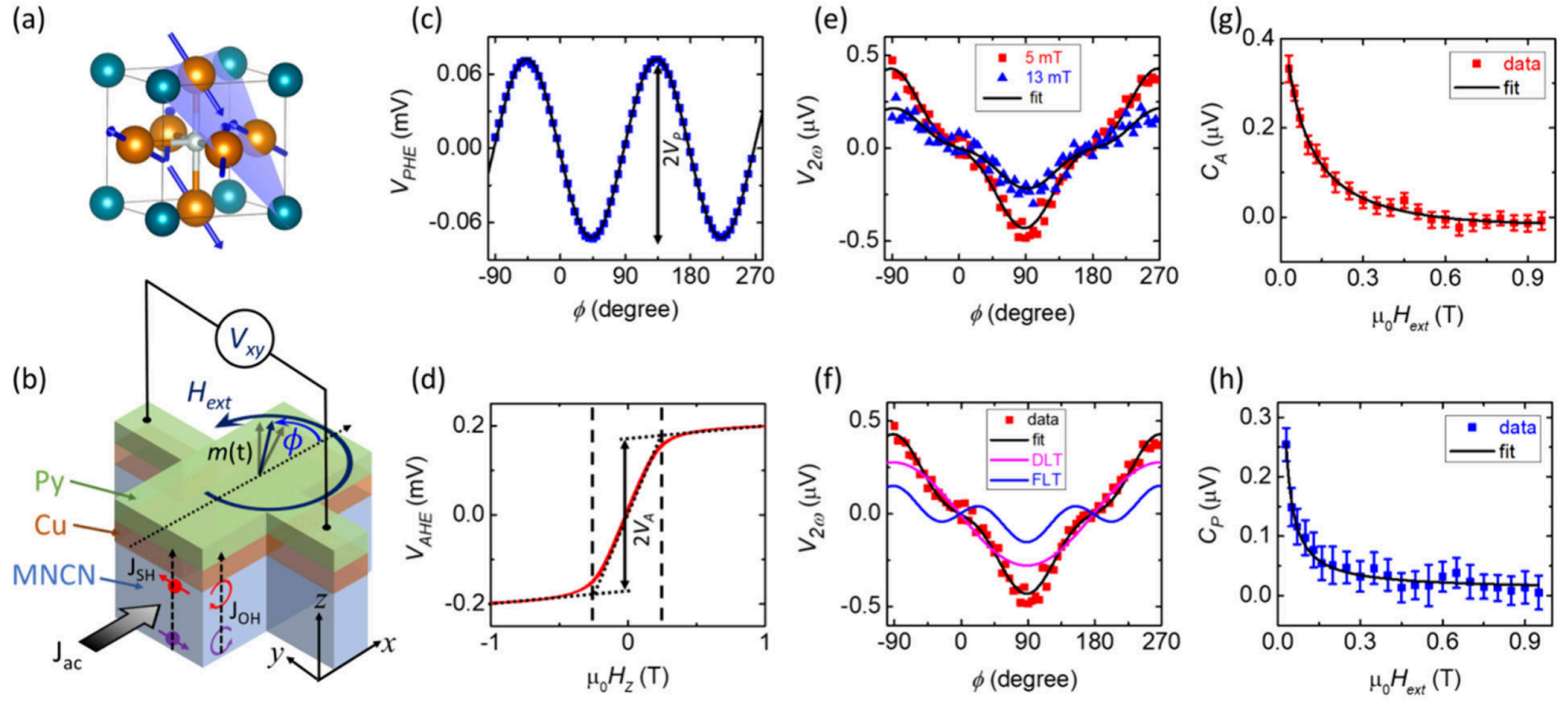
Second harmonic Hall (SHH) measurements. (a) The crystal structure of Mn_3_Ni_0.35_Cu_0.65_N (MNCN) with the spins in the Γ_4*G*_ configuration. (b) Schematic representation of SHH measurements. Measured first harmonic voltages (*V*_1ω_) by rotating the magnetic field (*H*_*ext*_) in the plane to determine *V*_*P*_ (c) and sweeping the magnetic field out of the plane to determine *V*_*A*_ and *H*_⊥_ (d). (e) Measured second harmonic Hall voltage (*V*_2ω_) signal for two different values of *H*_*ext*_. (f) Fitting of the experimental data (red squares) with the contribution from DLT (pink curve) and FLT (blue curve). (g, h) Extracted values of *C*_*A*_ and *C*_*P*_ as a function of *H*_*ext*_.

To quantify SOTs, we adopt the well-established technique of second harmonic Hall (SHH) measurements^37–41^ performed in a three-dimensional vector-cryo setup in the presence of an external magnetic field (*H*_*ext*_) at different temperatures (*T*) ranging from 4 to 300 K. The schematics of the experiment are shown in Figure 1b. We apply a low frequency (613 Hz) alternating electric current (ac) and record both the first harmonic (*V*_1ω_) and second harmonic Hall (*V*_2ω_) voltages while rotating *H*_*ext*_ of different strengths (0.03 to 0.9 T), well above the in-plane saturation field of Py (<0.005 T). MNCN produces two different types of torques, i.e., (1) in-plane damping-like torque (DLT), ***τ*_*DL*_** ∝ ***m*** × (**σ** × ***m***), where ***m*** is the unit vector of Py magnetization and **σ** is generated by angular momentum from SHE and/or OHE. For the DLT, the equivalent current-induced effective spin–orbit field (SOF), ***H***_***DL***_^***Z***^, deflects the magnet out of the plane, creating an oscillation in the resistance due to AHE. (2) Out-of-plane field-like torque (FLT), ***τ*_*FL*_** ∝ ***m*** × (***H***_***FL***_^***Oe***^ + ***H***_***FL***_^***Y***^), where ***H*_*Oe*_** is the Oersted field generated by the conductive shunting layers (e.g., Cu and MNCN) and ***H***_***FL***_^***Y***^ corresponds to interfacial SOF^41^ which is found to be negligible and not the main focus of this work. FLT from ***H***_***FL***_^***Oe***^ deflects the magnet in the sample plane, resulting in oscillating resistance due to the planar Hall effect (PHE). The mixing of the oscillating applied current and oscillating resistance produces *V*_2ω_, which can be expressed by eq 1 ^37–41^ in the absence of any other unconventional torques.

1 $$V_{2\omega }=C_{A}\;\cos\phi +C_{P}\;\cos\phi \;\cos2\phi$$

where

2 $$\{\begin{array}{l} C_{P}=-\left(H_{FL}^{Oe}+H_{FL}^{Y}\right)\frac{V_{P}}{H_{ext}}+C_{0}\\ C_{A}=-H_{DL}^{Z}\frac{V_{A}}{2\left(H_{ext}+H_{⊥}\right)}+V_{ANE}+V_{ONE}H_{ext} \end{array}$$

*V*_*ANE*_ and *V*_*ONE*_ are the strength of anomalous Nernst effect (ANE) and ordinary Nernst effect (ONE), respectively, which are generated due to the unintentional out-of-plane thermal gradient that is coupled to the in-plane magnetization of Py and *H*_*ext*_ respectively.^39^ *V*_*P*_ is the coefficient of the PHE voltage, *V*_*PHE*_ = *V*_*P*_ sin 2ϕ, where ϕ is the angle between applied current and magnetization in the sample plane (*xy*-plane) (Figure 1b). *V*_*P*_ is determined by measuring *V*_1ω_ while rotating *H*_*ext*_ in the plane (Figure 1c). *V*_*A*_ is the coefficient of the AHE voltage, *V*_*AHE*_ = *V*_*A*_ cos θ, where θ is the angle between the magnetization and out-of-plane *z*-axis (Figure 1d). The coefficient *V*_*A*_ is obtained from *V*_1ω_ while sweeping *H*_*ext*_ out of the plane (Figure 1d). The same measurement also provides us with the information on *H*_⊥_, the out-of-plane demagnetization field, which is in the range of 0.4 T for a 4 nm thick Py film (Figure 1d). More details can be found in the Supporting Information.

Figure 1e shows a typical second harmonic voltage (*V*_2ω_) measured in our experiment for two different values of *H*_*ext*_, 0.05 T (red squares) and 0.13 T (blue triangles), which are fit to eq 1 (black curves). We find that the magnitude of the measured *V*_2ω_ decreases for higher values of *H*_*ext*_, suggesting that the signals predominantly originate from the SOTs (eqs 1 and 2). Unlike other similar types of single crystal AFMs, we do not observe any large contribution of unconventional torques^17,42^ in our Γ_4g_ configuration, which could be due to the presence of domain variants. Figure 1f shows the fitting components of *V*_2ω_ for *H*_*ext*_ = 0.05 T using eq 1. The cos ϕ component (pink curve) indicates a dominant contribution from DLT, whereas the cos ϕ cos 2ϕ component (blue curve) indicates an FLT. To quantify the current-induced spin–orbit fields, *H*_*DL*_^*Z*^ and *H*_*FL*_^*Y*^, we plot these fitting coefficients, *C*_*A*_ (red squares) and *C*_*P*_ (blue squares) as a function of *H*_*ext*_ which fit well to eq 2 (black curve in Figure 1e,f). We find that both *C*_*A*_ and *C*_*P*_ decrease for higher magnitude of *H*_*ext*_ and saturate close to zero, suggesting SOTs are the dominant source of the *V*_2ω_ signal and thermal signals, e.g., ANE and ONE are negligible. Note that, it is very important to perform the in-plane SHH measurement by rotating *H*_*ext*_ up to a high value (*H*_*ext*_ = 0.9 T), much larger than the demagnetization field (*H*_⊥_ ∼ 0.4 T in our case), to get an accurate estimation of *H*_*DL*_^*Z*^.^39^ The DLT efficiency per unit electric field (ξ_*DL*_^*E*^) and per unit current density (ξ_*DL*_^*j*^) can be obtained from eq 3:

3 $$\{\begin{array}{l} \xi_{DL}^{E}=-\frac{2e}{\hbar }\mu_{0}M_{S}t_{FM}\frac{H_{DL}^{Z}}{E}\\ \xi_{DL}^{j}=\xi_{DL}^{E}\rho \end{array}$$

where μ_0_ is the vacuum permeability, *ℏ* is Planck’s constant, *e* is the electronic charge, *M*_*S*_ is the saturation magnetization as obtained from the SQUID magnetometry (600 emu/cc ≈ 0.75 T), and ρ is the electrical resistivity of MNCN (Figure 3a). ξ_*DL*_^*E*^ and ξ_*DL*_^*j*^ are lower bounds of the internal spin-Hall conductivity (*σ*_*SH*_) and spin-Hall angle (*θ*_*SH*_) respectively, due to the losses of the angular momentum transfer at the interface. To understand the origins of the SOTs, we next probe its temperature dependence.

MNCN exhibits a metallic behavior showing an increase in the resistivity (ρ) with an increase of the temperature (Figure 2a). There is a prominent change in ρ around 210 K, suggesting a transition from the antiferromagnetic to paramagnetic phase, which is consistent with the literature.^10,11,36^ Figure 2b,c show a strong and unusual variation of ξ_*DL*_^*E*^ (black squares) with respect to both temperature (*T*) and longitudinal electrical conductivity (*σ*_*xx*_). There is a large enhancement in both ξ_*DL*_^*E*^ (nearly 70%) and ξ_*DL*_^*j*^ (approximately 140%) near *T*_*N*_ (Figure 2b-d), strongly suggesting an influence of spin fluctuations in our NC-AFM. The maximum ascertained values of $\xi_{DL}^{E}=\frac{\hbar }{2e}$ ­(1.2 ± 0.1) × 10^5^ S/m and ξ_*DL*_^*j*^ = 0.30 ± 0.03 are much larger than those of the commonly studied heavy metals such as Pt^43^ (ξ_*DL*_^*j*^ = 0.07) and, most surprisingly, in the absence of any heavy element in the MNCN compound. As detailed in the Supporting Information, we have verified that the self-induced torques are negligible by conducting SHH measurements in Cu/Py/Cu samples. We have further performed spin-torque ferromagnetic resonance (ST-FMR)^42–44^ measurements at room temperature, which is consistent with SHH measurements, confirming a strong SOT generated by MNCN (for details, see the Supporting Information).

**Figure 2:**
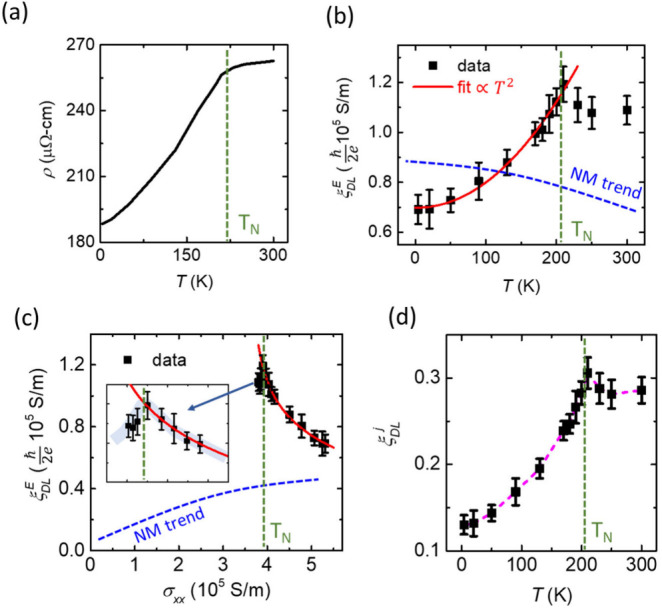
Temperature dependence of SOTs. (a) Resistivity (ρ) of MNCN as a function of temperature (*T*). The slope change of resistivity around 210 K indicates the Néel temperature (*T*_*N*_). Damping-like torque (DLT) efficiency per unit electric field, ξ_*DL*_^*E*^, (black squares) as a function of *T* (b) and electrical conductivity, *σ*_*xx*_ (c). The blue-dashed curve in panels (b) and (c) represents the expected dependence of ξ_*DL*_^*E*^ for a conventional nonmagnet (NM), such as Pt. The solid red curve represents the fitting of ξ_*DL*_^*E*^ with respect to the temperature or *σ*_*xx*_. The inset in (c) is a zoomed-in image near the transition temperature. (d) Estimated DLT efficiency per unit current density, ξ_*DL*_^*j*^, as a function of temperature (*T*).

The general trend of ξ_*DL*_^*E*^ and *σ*_*xx*_ as a function of temperature for a typical nonmagnet (such as Pt) is shown by the blue dashed line in Figure 2b,c.^3,45,46^ For the commonly used nonmagnets, we expect roughly σ_*DL*_^*E*^ ∝ *σ*_*xx*_ in the “dirty metal regime” (for *σ*_*xx*_ < 10^6^ S/m) due to the reduction of the carrier lifetime when the mean free path becomes comparable to the lattice constants.^3,45,46^ As the temperature is lowered, when *σ*_*xx*_ increases to the range of 10^6^–10^8^ S/m (“clean metal regime”), σ_*DL*_^*E*^ becomes nearly independent of *σ*_*xx*_ as the carrier lifetime becomes comparable to the SOC energy.^3,45,46^ For the ultraclean metals (*σ*_*xx*_ > 10^8^ S/m), when other spin-independent scattering processes are greatly suppressed, we can observe the contribution from the extrinsic spin-dependent scattering potentials producing σ_*DL*_^*E*^ ∝ σ_*xx*_^*n*^ where *n* > 1.^3,45^ Below the Néel temperature (*T*_*N*_), in our case, σ_*DL*_^*E*^ decreases as *σ*_*xx*_ increases with the decrease in temperature (Figure 2b,c), which is odd with any of these proposed mechanisms.

To ascertain possible origins of our observed large DLT efficiency, we carried out density functional theory (DFT) calculations of MNCN in both the nonmagnetic phase and NC-AFM phase with the static “all in-all-out” magnetic configuration (Figure 3a,b), followed by atomistic spin dynamics simulations (Figure 3c). For both magnetic and nonmagnetic phases, the DFT calculation predicts a small intrinsic spin-Hall conductivity (*σ*_*SH*_) below $-\frac{e}{\hbar }$ 1 × 10^4^ S/m with a negative sign (similar to Ta), and a significant orbital-Hall conductivity $\sigma_{OH}∼+\frac{e}{\hbar }$ 5 × 10^5^ S/m with a positive sign (similar to Pt). The spin–orbit coupling is very small in this material and does not contribute to OHE. Note that the magnitude of *σ*_*OH*_ slightly decreases in the magnetic phase compared to the nonmagnetic phase, suggesting that OHE fundamentally originates from the material’s crystal structure.

**Figure 3:**
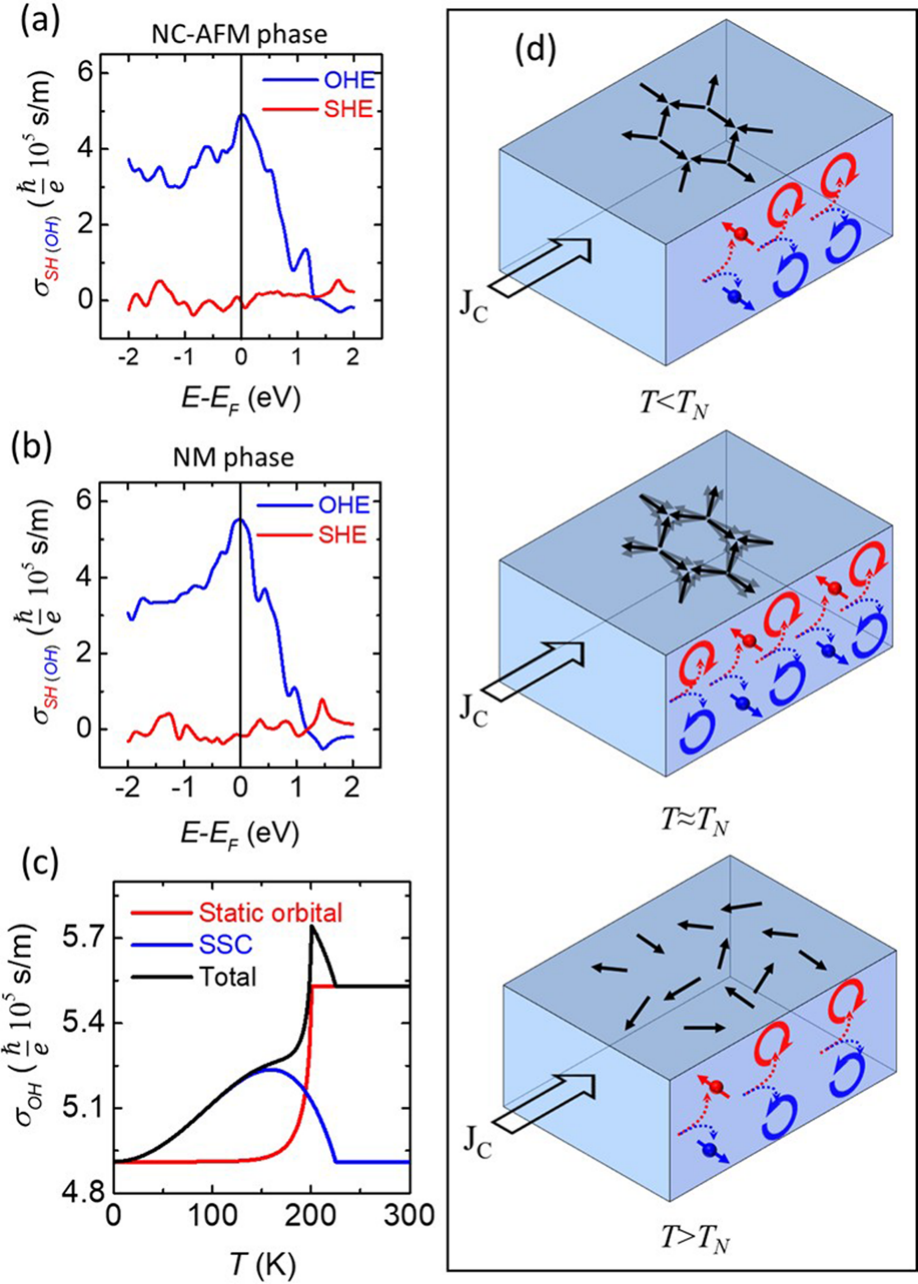
Origins of spin–orbital torques. DFT calculations of spin-Hall conductivity (*σ*_*SH*_) and orbital-Hall conductivity (*σ*_*OH*_) of MNCN in NC-AFM and NM phases in panels (a) and (b), respectively. (c) The interplay between scalar spin chirality (SSC) induced (blue) and static mean-field induced OHE (red) predicting anomalous temperature dependence of the resultant *σ*_*OH*_ (black curve). The blue curve in panel (c) represents SSC, which exhibits *σ*_*OH*_ = 0 at 0 K, and has been shifted vertically for clarity in comparison. (d) Schematic representation of the generation of spin-current (arrow with a solid ball) and orbital current (semicircular arrow) across different temperature regimes.

Our reported value of $\xi_{DL}^{E}=+\frac{e}{2\hbar }$ ­(1.2 ± 0.1) × 10^5^ S/m is much larger than the predicted value of *σ*_*SH*_ with opposite sign. We point out that such a sign change in SOT measurements is a key signature of OHE, as previously identified for other systems in the literature.^44,47,48^ Therefore, we attribute the large and positive sign of ξ_*DL*_^*E*^ observed in MNCN/Ni_81_Fe_19_ bilayers in the nonmagnetic phase (above *T*_*N*_) to the OHE, reporting the first experimental demonstration of OHE in any NC-AFM. The generated orbital current from MNCN is converted into the spin current in the magnet using the SOC of Ni, which constitutes of 81% Py, thereby generating large orbital torques. However, the strong temperature dependence of SOTs below *T*_*N*_ cannot be fully explained with this intrinsic OHE from the orbitals, as it is necessary to consider the role of chirality and extrinsic scattering arising from noncollinear magnetic ordering at finite temperature due to spin fluctuations. The generation of SOT has been studied in various NC-AFMs, including IrMn_3_, Mn_3_GaN, Mn_3_Sn, and Mn_3_Pt, with reported SOT efficiencies ranging from 0.1 to 0.3.^17,18,49–55^ While our estimated SOT efficiency (0.3) is among the highest, the most striking observation is its significant enhancement near *T*_*N*_, suggesting a novel mechanism for harnessing orbital current in NC-AFMs.

The observed temperature dependence of SOTs shows qualitative agreement with previous reports in ferromagnets^29–31^ and collinear AFMs,^27,32^ which was attributed to extrinsic scattering mechanisms driven by magnetic fluctuations. Ref 14 has theoretically studied the chirality-induced spin current in NC-AFMs, which can also qualitatively explain our results considering the competition of this chirality-induced spin current and previously explained orbital current, particularly applicable below *T*_*N*_. It is noteworthy that in our case ξ_*DL*_^*E*^ ∝ *T* ^2^ below *T*_*N*_, whereas it exhibits a more pronounced effect with sharp peaks around the transition temperature for ferromagnets^29–31^ and collinear antiferromagnets.^27,56^ In addition to the existing scattering-driven processes, we propose an alternative mechanism, the chirality-induced orbital current generation influenced by spin fluctuation across and below *T*_*N*_, which could also play an important role in our NC-AFM. This conjecture aligns with the recent theoretical prediction^20^ and experimental demonstration^21^ of orbital magnetism by magnonic excitations. Therefore, this phenomenon can also exist in other types of NC-AFM.

We expand the possibility of the temperature-dependent OHE upon the framework introduced in ref 20 for MNCN. We observe that the scalar spin chirality (SSC) resulting from fluctuating spins increases up to *T*_*N*_, followed by a rapid decline to zero after *T*_*N*_ (blue curve in Figure 3c), coming hand-in-hand with chirality-induced orbital Hall current. Note that SSC would produce σ_*OH*_ = 0 at 0 K and this curve has been shifted vertically for clarity. The red curve in Figure 3c illustrates the mean-field OHE variation originating from the suppression of magnetic order across the magnetic phase, bridging the limiting cases of *T* = 0 and *T* > *T*_*N*_. Its functional form lies in the assumption of the direct proportionality to the order parameter’s temperature dependence (see Section S5 and eq S11 in the Supporting Information for more details). The interplay between these processes may generate a peak in the magnitude of the orbital-Hall current, as elucidated by the black curve in Figure 3c suggesting a qualitative agreement with our experiments (Figure 2c). It is important to note that the predicted OHE from scalar spin chirality is expected to be proportional to *T* ^2^ (see Section S5 in the Supporting Information) for low temperature, which agrees with our experimental results shown in Figure 2b. Notably, in our material, M_3_Ni_0.35_Cu_0.65_N, the Γ_4g_ phase is stabilized with spins oriented in an all-in-all-out configuration (Figure 1a). Further material optimization is required to achieve the Γ_5g_ phase, which can exhibit different chiralities. Comparing the SOT generation in these two phases with the chirality dependence would be intriguing and remains an exciting direction for future research. This scenario potentially unveils an innovative mechanism to enhance orbital currents through fluctuating spins, offering a new perspective on the significance of scalar spin chirality in the transport properties of broad categories of the noncollinear antiferromagnets. The proposed new mechanism can coexist with the scattering-based spin and orbital current generation processes.

In conclusion, we report an unprecedented temperature dependence of the strong spin–orbit torques in the epitaxial noncollinear thin film antiferromagnet Mn_3_Ni_0.35_Cu_0.65_N, which peaks around the Néel temperature (∼210 K) with estimated spin torque efficiency per unit current density (ξ_*DL*_^*j*^) approximately 0.3, significantly larger than that can be realized using conventional heavy metals such as Pt. Our experimental observation strongly suggests a dominant contribution from the orbital-Hall effect above the transition temperature, which agrees with the density functional theory calculation in terms of sign and also in magnitude. The strong temperature dependence of torques around and below Néel temperature could be explained by both extrinsic skew-scattering driven and chirality-induced spin and orbital currents triggered by spin fluctuations. These concepts are exciting avenue for future research and can open up new prospects for MRAM applications by achieving large spin–orbit torques.

## Supplementary Material



## Data Availability

The data that support the findings of this study are available from the corresponding author upon reasonable request.

## Notes

Sample preparation and characterization; details of SHH measurement technique; comparison with ST-FMR data MNCN/Cu/Py/Cu/cap at room temperature; SHH data of Cu/Py/Cu samples; and computational details (PDF)

## ¶

Department of Physics, University of Kashmir, Hazratbal, Srinagar, 190006,Jammu and Kashmir, India

## References

[1] Manchon A., Železný J., Miron I. M., Jungwirth T., Sinova J., Thiaville A., Garello K., Gambardella P. (2019). Current-Induced
Spin-Orbit Torques in Ferromagnetic and Antiferromagnetic Systems. Rev. Mod. Phys..

[2] Dieny B., Prejbeanu I. L., Garello K., Gambardella P., Freitas P., Lehndorff R., Raberg W., Ebels U., Demokritov S. O., Akerman J., Deac A., Pirro P., Adelmann C., Anane A., Chumak A. V., Hirohata A., Mangin S., Valenzuela S. O., Onbaşlı M. C., D’Aquino M., Prenat G., Finocchio G., Lopez-Diaz L., Chantrell R., Chubykalo-Fesenko O., Bortolotti P. (2020). Opportunities and Challenges for Spintronics in the
Microelectronics Industry. Nat. Electron..

[3] Sinova J., Valenzuela S. O., Wunderlich J., Back C. H., Jungwirth T. (2015). Spin Hall
Effects. Rev. Mod. Phys..

[4] Go D., Jo D., Lee H.-W., Kläui M., Mokrousov Y. (2021). Orbitronics:
Orbital Currents in Solids. Europhys. Lett..

[5] Gupta R., Bouard C., Kammerbauer F., Ledesma-Martin J. O., Bose A., Kononenko I., Martin S., Usé P., Jakob G., Drouard M., Kläui M. (2025). Harnessing
Orbital Hall Effect in Spin-Orbit Torque MRAM. Nat. Commun..

[6] Chen H., Qin P., Yan H., Feng Z., Zhou X., Wang X., Meng Z., Liu L., Liu Z. (2022). Noncollinear Antiferromagnetic
Spintronics. Materials Lab.

[7] Chen H., Niu Q., MacDonald A. H. (2014). Anomalous Hall Effect Arising from Noncollinear Antiferromagnetism. Phys. Rev. Lett..

[8] Nakatsuji S., Kiyohara N., Higo T. (2015). Large Anomalous
Hall Effect in a
Non-Collinear Antiferromagnet at Room Temperature. Nature.

[9] Nayak A. K., Fischer J. E., Sun Y., Yan B., Karel J., Komarek A. C., Shekhar C., Kumar N., Schnelle W., Kübler J., Felser C., Parkin S. S. P. (2016). Large
Anomalous
Hall Effect Driven by a Nonvanishing Berry Curvature in the Noncolinear
Antiferromagnet Mn_3_Ge. Sci. Adv..

[10] Zhao K., Hajiri T., Chen H., Miki R., Asano H., Gegenwart P. (2019). Anomalous
Hall Effect in the Noncollinear Antiferromagnetic
Antiperovskite Mn_3_Ni_1‑x_Cu_x_N. Phys. Rev. B.

[11] Rajan A., Saunderson T. G., Lux F. R., Díaz R. Y., Abdullah H. M., Bose A., Bednarz B., Kim J.-Y., Go D., Hajiri T., Shukla G., Gomonay O., Yao Y., Feng W., Asano H., Schwingenschlögl U., López-Díaz L., Sinova J., Mokrousov Y., Manchon A., Kläui M. (2023). Revealing
the Higher-Order Spin Nature
of the Hall Effect in Non-Collinear Antiferromagnet Mn_3_Ni_0.35_Cu_0.65_N. arXiv.

[12] Železný J., Zhang Y., Felser C., Yan B. (2017). Spin-Polarized Current
in Noncollinear Antiferromagnets. Phys. Rev.
Lett..

[13] Zhang Y., Železný J., Sun Y., van den
Brink J., Yan B. (2018). Spin Hall Effect Emerging from a
Noncollinear Magnetic Lattice without Spin-Orbit Coupling. New J. Phys..

[14] Ishizuka H., Nagaosa N. (2018). Spin Chirality Induced Skew Scattering
and Anomalous
Hall Effect in Chiral Magnets. Sci. Adv..

[15] Chen X., Higo T., Tanaka K., Nomoto T., Tsai H., Idzuchi H., Shiga M., Sakamoto S., Ando R., Kosaki H., Matsuo T., Nishio-Hamane D., Arita R., Miwa S., Nakatsuji S. (2023). Octupole-Driven
Magnetoresistance in an Antiferromagnetic Tunnel Junction. Nature.

[16] Qin P., Yan H., Wang X., Chen H., Meng Z., Dong J., Zhu M., Cai J., Feng Z., Zhou X., Liu L., Zhang T., Zeng Z., Zhang J., Jiang C., Liu Z. (2023). Room-Temperature
Magnetoresistance in an All-Antiferromagnetic Tunnel
Junction. Nature.

[17] Nan T., Quintela C. X., Irwin J., Gurung G., Shao D. F., Gibbons J., Campbell N., Song K., Choi S.-Y., Guo L., Johnson R. D., Manuel P., Chopdekar R. V., Hallsteinsen I., Tybell T., Ryan P. J., Kim J. W., Choi Y., Radaelli P. G., Ralph D. C., Tsymbal E. Y., Rzchowski M. S., Eom C. B. (2020). Controlling Spin Current Polarization
through Non-Collinear Antiferromagnetism. Nat.
Commun..

[18] Dc M., Shao D. F., Hou V. D. H., Vailionis A., Quarterman P., Habiboglu A., Venuti M. B., Xue F., Huang Y. L., Lee C. M., Miura M., Kirby B., Bi C., Li X., Deng Y., Lin S. J., Tsai W., Eley S., Wang W. G., Borchers J. A., Tsymbal E. Y., Wang S. X. (2023). Observation
of Anti-Damping Spin-Orbit Torques Generated
by in-Plane and out-of-Plane Spin Polarizations in MnPd_3_. Nat. Mater..

[19] Kimata M., Chen H., Kondou K., Sugimoto S., Muduli P. K., Ikhlas M., Omori Y., Tomita T., MacDonald A. H., Nakatsuji S., Otani Y. (2019). Magnetic and Magnetic Inverse Spin
Hall Effects in a Non-Collinear Antiferromagnet. Nature.

[20] Zhang L., Go D., Hanke J.-P., Buhl P. M., Grytsiuk S., Blügel S., Lux F. R., Mokrousov Y. (2020). Imprinting
and Driving Electronic
Orbital Magnetism Using Magnons. Commun. Phys..

[21] Alahmed L., Zhang X., Wen J., Xiong Y., Li Y., Zhang L., Lux F., Freimuth F., Mahdi M., Mokrousov Y., Novosad V., Kwok W.-K., Yu D., Zhang W., Lee Y. S., Li P. (2022). Evidence of Magnon-Mediated
Orbital Magnetism in a Quasi-2D Topological Magnon Insulator. Nano Lett..

[22] Xiao D., Chang M.-C., Niu Q. (2010). Berry Phase
Effects on Electronic
Properties. Rev. Mod. Phys..

[23] Tanaka T., Kontani H., Naito M., Naito T., Hirashima D. S., Yamada K., Inoue J. (2008). Intrinsic
Spin Hall Effect and Orbital
Hall Effect in 4d and 5d Transition Metals. Phys. Rev. B.

[24] Guo G. Y., Murakami S., Chen T.-W., Nagaosa N. (2008). Intrinsic Spin Hall
Effect in Platinum: First-Principles Calculations. Phys. Rev. Lett..

[25] Freimuth F., Blügel S., Mokrousov Y. (2010). Anisotropic Spin Hall Effect from
First Principles. Phys. Rev. Lett..

[26] Okamoto S., Egami T., Nagaosa N. (2019). Critical Spin
Fluctuation Mechanism
for the Spin Hall Effect. Phys. Rev. Lett..

[27] Fang C., Wan C., Zhang X., Okamoto S., Ma T., Qin J., Wang X., Guo C., Dong J., Yu G., Wen Z., Tang N., Parkin S. S. P., Nagaosa N., Lu Y., Han X. (2023). Observation
of the Fluctuation Spin Hall Effect in a Low-Resistivity
Antiferromagnet. Nano Lett..

[28] Crépieux A., Bruno P. (2001). Theory of the Anomalous
Hall Effect from the Kubo Formula and the
Dirac Equation. Phys. Rev. B.

[29] Wei D. H., Niimi Y., Gu B., Ziman T., Maekawa S., Otani Y. (2012). The Spin Hall Effect
as a Probe of Nonlinear Spin Fluctuations. Nat.
Commun..

[30] Ou Y., Ralph D. C., Buhrman R. A. (2018). Strong
Enhancement of the Spin Hall
Effect by Spin Fluctuations near the Curie Point of FexPt1-x Alloys. Phys. Rev. Lett..

[31] Wu P.-H., Qu D., Tu Y.-C., Lin Y.-Z., Chien C. L., Huang S.-Y. (2022). Exploiting
Spin Fluctuations for Enhanced Pure Spin Current. Phys. Rev. Lett..

[32] Frangou L., Oyarzún S., Auffret S., Vila L., Gambarelli S., Baltz V. (2016). Enhanced Spin Pumping Efficiency in Antiferromagnetic IrMn Thin Films
around the Magnetic Phase Transition. Phys.
Rev. Lett..

[33] Qiu Z., Li J., Hou D., Arenholz E., N’Diaye A. T., Tan A., Uchida K., Sato K., Okamoto S., Tserkovnyak Y., Qiu Z. Q., Saitoh E. (2016). Spin-Current Probe for Phase Transition
in an Insulator. Nat. Commun..

[34] Hou D., Qiu Z., Barker J., Sato K., Yamamoto K., Vélez S., Gomez-Perez J. M., Hueso L. E., Casanova F., Saitoh E. (2017). Tunable Sign
Change of Spin Hall Magnetoresistance in Pt/NiO/YIG Structures. Phys. Rev. Lett..

[35] Qiu Z., Hou D., Barker J., Yamamoto K., Gomonay O., Saitoh E. (2018). Spin Colossal
Magnetoresistance in an Antiferromagnetic Insulator. Nat. Mater..

[36] Miki R., Zhao K., Hajiri T., Gegenwart P., Asano H. (2020). Epitaxial Growth and Orientation-Dependent Anomalous Hall Effect
of Noncollinear Antiferromagnetic Mn_3_Ni_0.35_Cu_0.65_N Films. J. Appl. Phys..

[37] Hayashi M., Kim J., Yamanouchi M., Ohno H. (2014). Quantitative Characterization of
the Spin-Orbit Torque Using Harmonic Hall Voltage Measurements. Phys. Rev. B.

[38] Avci C. O., Garello K., Gabureac M., Ghosh A., Fuhrer A., Alvarado S. F., Gambardella P. (2014). Interplay
of Spin-Orbit Torque and
Thermoelectric Effects in Ferromagnet/Normal-Metal Bilayers. Phys. Rev. B.

[39] Roschewsky N., Walker E. S., Gowtham P., Muschinske S., Hellman F., Bank S. R., Salahuddin S. (2019). Spin-Orbit
Torque and Nernst Effect in Bi-Sb/Co Heterostructures. Phys. Rev. B.

[40] Schulz T., Lee K., Krüger B., Lo Conte R., Karnad G. V., Garcia K., Vila L., Ocker B., Ravelosona D., Kläui M. (2017). Effective
Field Analysis Using the Full Angular Spin-Orbit
Torque Magnetometry Dependence. Phys. Rev. B.

[41] Dutta S., Bose A., Tulapurkar A. A., Buhrman R. A., Ralph D. C. (2021). Interfacial
and Bulk Spin Hall Contributions to Fieldlike Spin-Orbit Torque Generated
by Iridium. Phys. Rev. B.

[42] Bose A., Schreiber N. J., Jain R., Shao D.-F., Nair H. P., Sun J., Zhang X. S., Muller D. A., Tsymbal E. Y., Schlom D. G., Ralph D. C. (2022). Tilted Spin Current Generated by the Collinear Antiferromagnet
Ruthenium Dioxide. Nat. Electron..

[43] Liu L., Moriyama T., Ralph D. C., Buhrman R. A. (2011). Spin-Torque Ferromagnetic
Resonance Induced by the Spin Hall Effect. Phys.
Rev. Lett..

[44] Bose A., Kammerbauer F., Gupta R., Go D., Mokrousov Y., Jakob G., Kläui M. (2023). Detection of Long-Range Orbital-Hall
Torques. Phys. Rev. B.

[45] Vignale G. (2010). Ten Years
of Spin Hall Effect. J. Supercond. Nov. Magn..

[46] Bose A., Nelson J. N., Zhang X. S., Jadaun P., Jain R., Schlom D. G., Ralph D. C., Muller D. A., Shen K. M., Buhrman R. A. (2020). Effects of Anisotropic Strain on Spin-Orbit Torque
Produced by the Dirac Nodal Line Semimetal IrO_2_. ACS Appl. Mater. Interfaces.

[47] Lee D., Go D., Park H.-J., Jeong W., Ko H.-W., Yun D., Jo D., Lee S., Go G., Oh J. H., Kim K.-J., Park B.-G., Min B.-C., Koo H. C., Lee H.-W., Lee O., Lee K.-J. (2021). Orbital Torque in Magnetic Bilayers. Nat. Commun..

[48] Hayashi H., Jo D., Go D., Gao T., Haku S., Mokrousov Y., Lee H.-W., Ando K. (2023). Observation
of Long-Range Orbital
Transport and Giant Orbital Torque. Commun.
Phys..

[49] Zhang W., Han W., Yang S., Sun Y., Zhang Y., Yan B., Parkin S. S. P. (2016). Giant Facet-Dependent
Spin-Orbit Torque and Spin Hall
Conductivity in the Triangular Antiferromagnet IrMn_3_. Science Advances..

[50] Kondou K., Chen H., Tomita T., Ikhlas M., Higo T., MacDonald A. H., Nakatsuji S., Otani Y. (2021). Giant Field-like Torque
by the out-of-Plane Magnetic Spin Hall Effect in a Topological Antiferromagnet. Nat. Commun..

[51] Bai H., Zhou X. F., Zhang H. W., Kong W. W., Liao L. Y., Feng X. Y., Chen X. Z., You Y. F., Zhou Y. J., Han L., Zhu W. X., Pan F., Fan X. L., Song C. (2021). Control of
Spin-Orbit Torques through Magnetic Symmetry in Differently Oriented
Noncollinear Antiferromagnetic Mn_3_Pt. Phys. Rev. B.

[52] Hu S., Shao D.-F., Yang H., Pan C., Fu Z., Tang M., Yang Y., Fan W., Zhou S., Tsymbal E. Y., Qiu X. (2022). Efficient Perpendicular Magnetization
Switching by a Magnetic Spin Hall Effect in a Noncollinear Antiferromagnet. Nat. Commun..

[53] Holanda J., Saglam H., Karakas V., Zang Z., Li Y., Divan R., Liu Y., Ozatay O., Novosad V., Pearson J. E., Hoffmann A. (2020). Magnetic Damping Modulation in IrMn_3_/Ni_80_Fe_20_ via the Magnetic Spin Hall
Effect. Phys. Rev. Lett..

[54] Bangar H., Khan K. I. A., Kumar A., Chowdhury N., Muduli P. K., Muduli P. K. (2023). Large Spin Hall
Conductivity in Epitaxial
Thin Films of Kagome Antiferromagnet Mn_3_Sn at Room Temperature. Adv. Quantum Technol..

[55] Kumar A., Gupta P., Chowdhury N., Khan K. I. A., Shashank U., Gupta S., Fukuma Y., Chaudhary S., Muduli P. K. (2023). Interfacial Origin of Unconventional
Spin-Orbit Torque
in Py/Γ-IrMn_3_. Adv. Quantum
Technol..

[56] Okamoto S., Nagaosa N. (2024). Critical Enhancement of the Spin Hall Effect by Spin
Fluctuations. npj Quantum Materials..

