# Supplementary material for: Fluctuation-Mediated Spin–Orbit Torque Enhancement in the Noncollinear Antiferromagnet Mn3Ni0.35Cu0.65N
Source: Nano Lett. 2025 May 13;25(20):8073–9. doi: 10.1021/acs.nanolett.4c05423 (PMC12100721; doi:10.1021/acs.nanolett.4c05423)
Supplement: Supplementary file 1 [file nl4c05423_si_001.pdf]

Supporting Information

Fluctuation-mediated spin-orbit torque enhancement in the noncollinear antiferromagnet $Mn_3Ni_{0.35}Cu_{0.65}N$

Arnab Bose[1,2*], Tom G. Saunderson[1,3], Aga Shahee[1$], Lichuan Zhang[4], Tetsuya Hajiri[5], Adithya Rajan[1], Durgesh Kumar[1], Dongwook Go[1,3], Hidefumi Asano[5], Udo Schwingenschlögl[6], Aurelien Manchon[7], Yuriy Mokrousov[1,3], Mathias Kläui[1,8#]

[1]*Institute of Physics, Johannes Gutenberg-University Mainz, Staudingerweg 7, Mainz, 55128, Germany*
[2]*Department of Electrical Engineering, Indian Institute of Technology, Kanpur, 201086, UP, India*
[3]*Peter Grünberg Institut and Institute for Advanced Simulation, Forschungszentrum Jülich and JARA, Jülich, 52425, Germany*
[4]*School of Physics and Electronic Engineering, Jiangsu University, Zhenjiang 212013, China*
[5]*Department of Materials Physics, Nagoya University, Nagoya, 464-8603, Japan*
[6]*Physical Science and Engineering Division, King Abdullah University of Science and Technology, Thuwal, 23955-6900, Saudi Arabia.*
[7]*Aix-Marseille Université, CNRS, CINaM, Marseille, 13009 France*
[8]*Centre for Quantum Spintronics, Norwegian University of Science and Technology, 7491 Trondheim, Norway*

S1. Sample preparation and device characterization

$Mn_3Ni_{0.35}Cu_{0.65}N$ (MNCN) films with a thickness of 15 nm are epitaxially grown on (111)-oriented single crystal MgO substrates using the reactive sputtering technique in the presence of Ar (96%) and N_2 (4%) gases at a partial pressure of 2 Pa. To achieve the epitaxial growth in the (111) orientation, MNCN films are annealed at 500°C. X-ray diffraction data for a MgO(111)/MNCN sample is shown in Fig. S1. The sharp peaks arise from the MgO (111) and (222) planes, while two intense peaks from MNCN (111) and (222) indicate epitaxial growth. Further details regarding the sample preparation and growth technique can be found in previous reports[1–3].

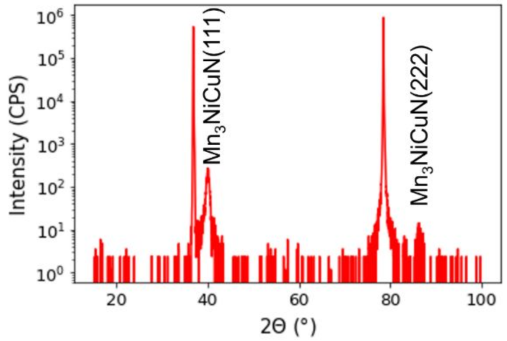

Figure S1: X-ray diffraction pattern on MgO(111)/$Mn_3Ni_{0.35}Cu_{0.65}N$ (MNCN) films.

The ϕ-scans in Fig. S2 show a 3-fold periodicity of the (220) peaks of MgO and MNCN, suggesting good crystalline alignment of the film and the substrate. Fig. S3(a) and (b) show the rocking curve of MNCN on (111) and (222) peaks, respectively. The finite spread of the peak suggests epitaxial growth with crystal defects, such as grain boundaries and dislocations, among others, which may be caused by lattice mismatch. However, it is noteworthy that the film and substrate are well aligned for magneto-transport measurements, which is the key property that is important for the purposes of this work.

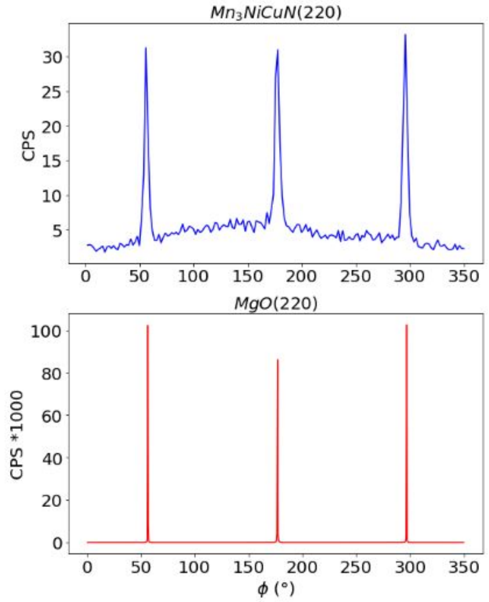

Figure S2: ϕ-scans of (220) planes of the MNCN thin film (top) and the MgO substrate (bottom) show the peaks with three-fold symmetry, demonstrating in-plane alignment of the thin film with the substrate.

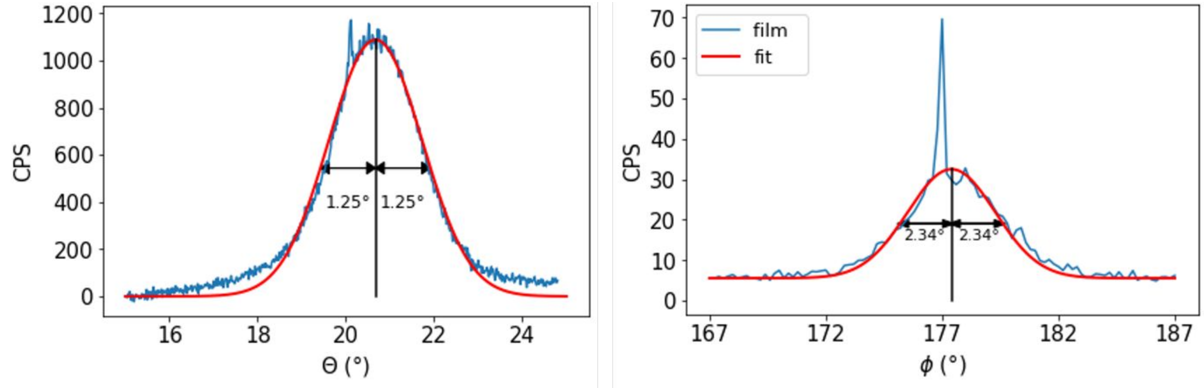

Figure S3: Rocking curves at the MNCN (111) peak (a) and MNCN (220) peak (b)

After the growth of the thin film, we proceed to pattern the devices into a Hall bar shape (50×20 μm^2), as illustrated schematically in Fig. S4a, using optical lithography, Ar-ion milling, and lift-off techniques. Following the patterning of the Hall bars, another round of optical lithography is performed, followed by lift-off, to create the contact pads using Cr (4 nm) / Au (80 nm). Prior to the deposition of the Cr/Au contacts, a mild Argon cleaning step is carried out to achieve transparent contacts. For the optical lithography, we use a bilayer resist, consisting of LOR-3B and S1813. Pattern development is done using MF-319 solution, while the lift-off process was performed in boiling PG-Remover. The samples undergo baking in the temperature range of 100°C to 135°C during these optical lithography and lift-off steps.

To measure the saturation magnetization (M_S) of the sample, we sweep an in-plane magnetic field (0.5 Tesla) within the sample plane (easy plane) at different temperatures in SQUID. The temperature dependence of M_S is shown in Fig. S4b. The M_S at room temperature is found to be approximately 600 emu/cc, slightly lower than that of bulk Py samples. However, M_S increases by approximately 10% at 4 K (Fig. S4b). Fig. S4c presents the temperature dependence of the longitudinal resistance of the stack (R_{xx}), which is typically measured by keeping a high resistor (20-100 kOhm) between the sample and the lock-in

amplifier so that it acts as a current source. The sudden change in R_{xx} around 210 K indicates the transition temperature of MNCN, which is consistent with the resistivity measurement of the bare MNCN film presented in the main paper (Fig. 2a).

A low-frequency alternating current (613 Hz) is sourced to the device using a lock-in amplifier (DSP Signal Recovery 7265) along the longitudinal direction (x-axis). The first and second harmonic Hall voltages ($V_{1\omega}$ and $V_{2\omega}$) are measured in the transverse direction using the standard lock-in detection technique. In the measurement, an external magnetic field (H_{ext}) is rotated within a range of 0.03 T to 0.9 T using a vector cryostat covering a temperature from 300 K to 4 K.

S2. Temperature-dependent second harmonic Hall (SHH) measurement

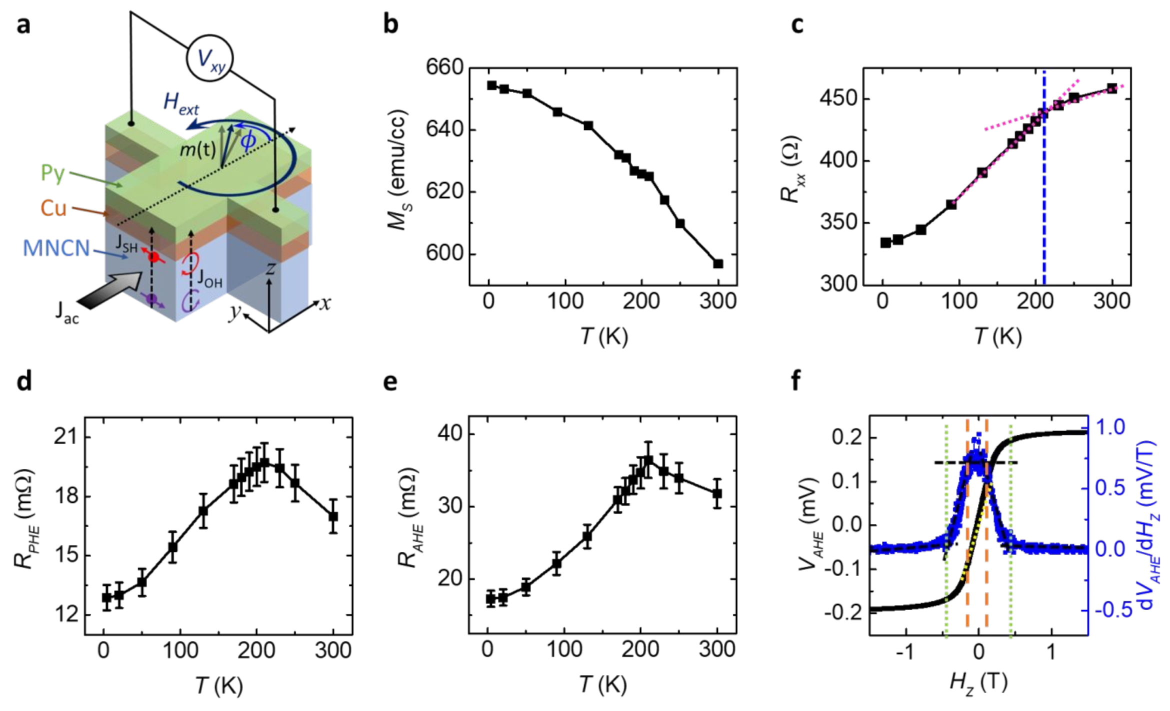

Fig. S4| Characterization of the device. **a**. Schematic representation of SHH measurement. **b.** Saturation magnetization (M_S) of Py as a function of temperature (T). **c.** Longitudinal resistance (R_{xx}) of the device as a function of temperature. Coefficient of the planar-Hall effect (PHE) (R_{PHE}) and anomalous Hall effect (R_{AHE}) of the whole stack as a function of temperature in panels **d** and **e,** respectively. **f**. Measurement of the first harmonic voltage (V_{AHE}) as a function of out-of-plane field sweep (H_Z) providing the data for AHE (black curve). Derivative of V_{AHE} with respect to H_Z is shown with the blue curve, which shows three distinct regimes, i.e. linear regime at low field values (H_Z < 0.14T), saturation regime at high fields ((H_Z > 0.14T)) and sublinear regime at intermediate fields.

The Hall resistance can be expressed as:

$$R_{1\omega}(\theta, \phi) = R_0 + R_{PHE}\sin 2\phi \sin^2\theta + R_{AHE}\cos\theta \quad \text{(s1)}$$

R_{PHE} corresponds to the coefficient of the planar-Hall effect (PHE), which can be quantified by measuring $V_{1\omega}$ while rotating a magnetic field (H_{ext}) in the sample plane, as depicted in Fig. 1c in the main text. The

temperature dependence of R_{PHE} is illustrated in Fig. S4d, which exhibits an enhancement near the transition temperature (T_N). This enhancement is further supported by the change in the slope of the longitudinal resistance (R_{xx}) observed in Fig. S4c and Fig. 2a. R_{AHE} is the coefficient of anomalous Hall effect (AHE), which can be determined by measuring $V_{1\omega}$ while sweeping H_{ext} out of the plane, as shown in Fig. S5d and Fig. 1d. We observe an enhancement of R_{AHE} near T_N (Fig. S5d) as well. The strong variation of R_{PHE} and R_{AHE} of the stack could be due to two possibilities: (1) The PHE and AHE of Py are influenced by the fluctuation of the adjacent antiferromagnetic (AFM) ordering of MNCN and (2) the spin-Hall magnetoresistance (SMR) due to the enhancement of spin-Hall effect (SHE) and orbital-Hall effect (OHE) of MNCN near T_N. Rigorous theoretical and experimental investigations are needed to understand its origin which is not the main focus of this work and kept as a future work.

Applied alternating current produces two different types of torques on the magnet, Py ($Ni_{80}Fe_{20}$)[4]:

(1) In-plane damping-like torque (DLT), $\tau_{DL} \propto m \times (\sigma_y \times m)$ where m is the unit vector of Py magnetization and σ is the direction of the generated angular momentum from SHE and/or OHE.

(2) Out-of-plane field-like torque (FLT), $\tau_{DL} \propto m \times \left(H_{FL}^{Oe} + H_{FL}^{Y}\right)$ where H_{FL}^{Oe} is the oersted field generated by the conductive shunting layers and H_{FL}^{Y}corresponds to interfacial spin-orbit fields (SOFs).

For DLT, the equivalent current induced effective SOF[5], H_{DL}^{Z}→$(\sigma_y \times m_{in-plane})$ deflects the magnet out of the plane (θ→$\theta + d\theta$), creating an oscillation in resistance due to AHE. FLT from H_{FL}^{Oe} deflects the magnet in the sample plane (ϕ→$\phi + d\phi$) resulting in an oscillating resistance due to PHE. So, we can expand R_{xy} as following.

$$R_{xy}(\theta + d\theta, \phi + d\phi) \approx R_{xy}(\theta,\phi) + \sum \frac{\partial R_{xy}}{\partial \theta} d\theta + \sum \frac{\partial R_{xy}}{\partial \phi} d\phi \tag{S5}$$

For the in-plane magnetized sample $\theta = 90$ degree at equilibrium. We can evaluate the resistance oscillations $\left(\frac{\partial R_{xy}}{\partial \theta}, \frac{\partial R_{xy}}{\partial \phi}\right)$ and deflections ($d\theta$,$d\phi$) as following:

$$\left(\frac{\partial R}{\partial \theta}\right)_{\theta=90^\circ} = -R_A \text{ and } \left(\frac{\partial R}{\partial \phi}\right)_{\theta=90^\circ,\,\phi} = 2R_P \cos 2\phi \tag{s3}$$

$$d\theta(t) \sim \frac{H_{DL}^{Z}\cos\phi}{H_{ext}+H_{\perp}} \sin\omega t \text{ and } d\phi(t) \sim \frac{\left(H_{FL}^{Oe}+H_{FL}^{Y}\right)\cos\phi}{H_{ext}} \sin\omega t \tag{s4}$$

$H_{\perp}$is the out of plane demagnetization field. The current induced SOFs also oscillate at the same frequency (ω) of the applied ac current, $I_o \sin\omega t$. The $\cos\phi$ term in Eqn (s3) arises due to the perpendicular component of the current induced effective fields on the magnetization. The rectified voltage due to this oscillating applied current and generated oscillating sample resistance can be expressed as:

$$V_{xy}(t) = R_{xy}(\theta + d\theta(t), \phi + d\phi(t))\, I_0 \sin\omega t$$

$$V_{xy}(t) = I_0/2(1 - \cos 2\omega t)\left[\frac{R_{AHE}\, H_{DL}^{Z}}{H_{ext}+H_{\perp}} + \frac{2R_{PHE}\left(H_{FL}^{Oe}+H_{FL}^{Y}\right)}{H_{ext}} \cos 2\phi\right]\cos\phi + I_0 \sin\omega t R(\theta,\phi) \tag{s5}$$

Equation (s5) predicts the generation of $V_{2\omega}$ with a 90-degree phase-shift which is experimentally measured using the locking detection technique. We can rewrite the equation (s5) for the second harmonic voltage expression ($V_{2\omega}$) considering the thermal signals as following.

$$V_{2\omega} = C_A \cos\phi + C_P \cos\phi \cos 2\phi \tag{s6}$$

Where $\begin{cases} C_P = -\left(H_{FL}^{Oe} + H_{FL}^{Y}\right)\frac{V_P}{H_{ext}} + C_0 \\ C_A = -H_{DL}^{Z}\frac{V_A}{2(H_{ext}+H_{\perp})} + V_{ANE} + V_{ONE}H_{ext} \end{cases}$ (s7)

V_{ANE} and V_{ONE} are the strength of anomalous Nernst effect (ANE) and ordinary Nernst effect (ONE) respectively, which are generated due to the unintentional out-of-plane thermal gradient (∇T_z) coupled to the in-plane magnetization of Py ($\nabla T_z \times M$) and H_{ext} ($\nabla T_z \times H_{ext}$) respectively[6].

Fig. 1e,f in the main paper shows a typical spectrum of $V_{2\omega}$ which is fit by equation (s6) to obtain the coefficients C_A and C_P from which we can estimate the SOFs for the damping-like torque (DLT) and field-like torque (FLT) respectively. We can quantify the DLT efficiency per unit electric field (ξ_{DL}^{E}) and per unit current density (ξ_{DL}^{j}) as follows:

$$\begin{cases} \xi_{DL}^{E} = -\frac{2e}{\hbar}\mu_0 M_S t_{FM}\frac{H_{DL}^{Z}}{E} \\ \xi_{DL}^{j} = \sigma_{DL}^{E}\rho \end{cases} \quad \text{(s8)}$$

μ_0 is the vacuum permeability, $\hbar$ is Planck's constant, e is the electronic charge, M_S is the saturation, ρ is the electrical resistivity of MNCN (Fig. 3a).

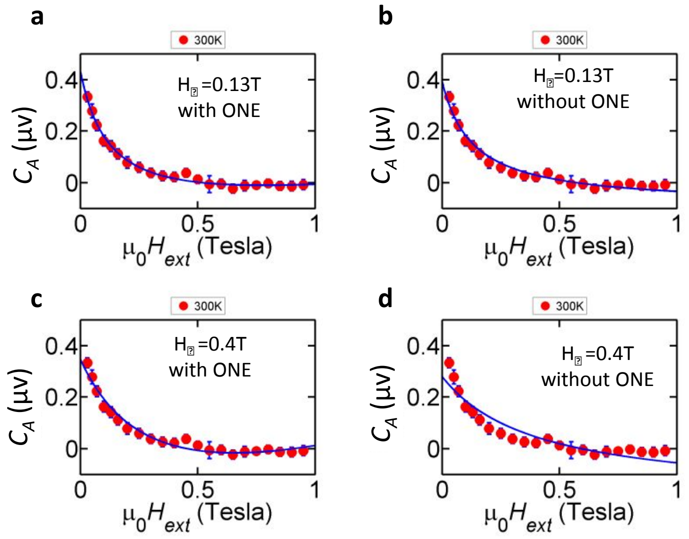

Fig. S5| Analysis of second harmonic Hall data. **a**, **b** represents coefficients, C_A (red circles) and the fit (blue curve) with respect to external field strength (H_{ext}) while considering with and without ordinary Nernst effect (ONE) respectively for $H_{\perp}$ = 0.13T. **c**, **d** shows the same for $H_{\perp}$ = 0.4T.

Fig. S5a-d show the extracted values of C_A as a function of H_{ext} at room temperature considering with and without the ONE term for two different values of $H_{\perp} = 0.13$ and 0.4 T. Fig. S5a,b show that the data can be fit with great confidence for $H_{\perp} = 0.13\ T$ where ONE has a little contribution. Note that 0.13 T is the edge of the linear region in V_{AHE} vs H_z curve (Fig. s1f). Note that the thermal signals are very small in our experiments since C_A goes very close to 0 at a high field while saturating. Hence, we expect a minor contribution from both ANE and ONE, as evident in Fig. S5a,b.

The experimentally measured $H_{\perp} \approx 0.4$ T, the required out-of-plane magnetic field to fully saturate Py out of plane (Fig. 1d and Fig. S5f). Surprisingly $H_{\perp} = 0.4$ T in fitting incurs a large deviation for both cases

considering with and without ONE (Fig. S5c,d). Without ONE, the poor agreement of the fitting can be easily seen in Fig. S5d. However, the fitting is slightly improved after including a significant effect of ONE (Fig. S5c) which is impractical in our measurements. From this we conclude that $H_{\perp} \approx 0.4$ T cannot be the effective demagnetization field that Py is experiencing when the spin-orbit fields are in the order few mT. This can be understood in Fig. S4e where we observe three distinct regimes in the R_{AHE} vs H_z curve i.e. (i) a linear region (H_{ext} <0.13T), (ii) saturation region (H_{ext}>0.4 T) and (iii) a sublinear region in between. One can appreciate it more by considering the blue curve in Fig. S5f representing dR_{AHE}/dH_Z. Our results clearly suggest that the magnet experiences effective demagnetization field, $H_{\perp}^{eff} \approx 0.13\ T$ which is much lower than the actual $H_{\perp} \approx 0.4\ T$ due to the sublinear behavior of R_{AHE} vs H_z curve. We point out that the reduced $H_{\perp}^{eff}$ is an important consideration in SHH measurements that might have unnoticed in majority of the previous works particularly for the cases where H_{ext} is smaller or comparable to $H_{\perp}$.

S3. Additional experimental results

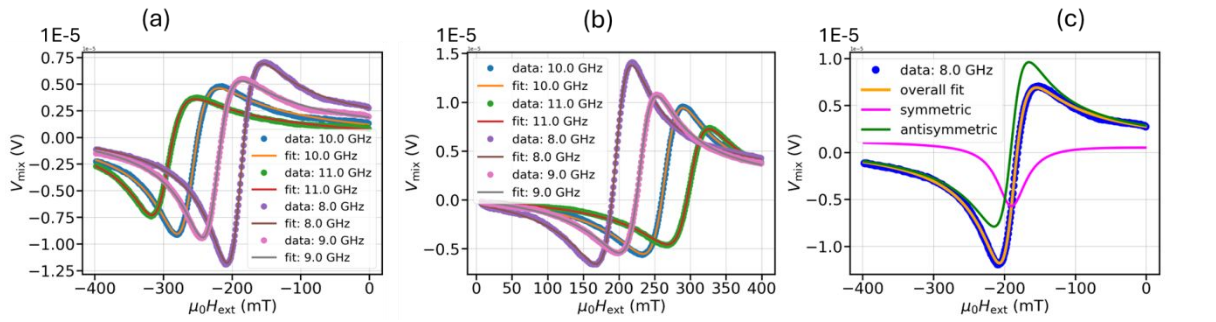

Figure S6. ST-FMR data for MNCN/Cu/Py/Cu/cap at room temperature. V_{mix} for the negative (a) and positive (b) field sweep at different frequencies. (c) Fitting of the V_{mix} with the symmetric (V_S) (pink curve) and anti-symmetric (V_A) (green) Lorentzian functions. Large V_S signifies substantial damping-like torques, consistent with SHH measurement as presented in the manuscript.

Spin-torque ferromagnetic resonance (ST-FMR) is the resonant analogue of the SHH measurement. In the ST-FMR technique, DC voltage is measured by sweeping a magnetic field at an angle ϕ (typically 45°) with respect to the applied radio frequency (RF) current. As discussed above, the RF current excites the magnet into resonance due to various current-induced torques (section S2). The homodyne mixing between the RF current and anisotropic magnetoresistance (AMR) produces a DC voltage spectrum (Fig. S6), which can be fitted to the symmetric (V_S) (pink curve in Fig. S6(c)) and anti-symmetric Lorentzian (V_A) functions

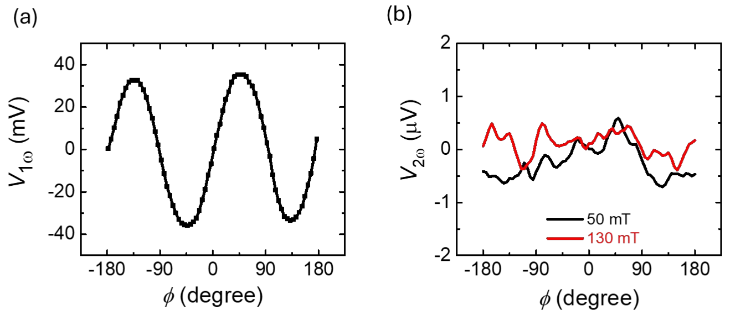

Figure S7. 1st and 2nd harmonic Hall voltages for SiO2/Ta(1)/Cu(2)/Py(5)/Cu(2)/MgO(1.5)/Ta(1.5)

(green curve in Fig. S6(c)). The sizable magnitude of V_S from the 15 nm thick MNCN source layer suggests a strong spin/orbital current generation, which is consistent with the SHH measurements.

We have measured the spin-orbit torque (SOT) using second-harmonic Hall (SHH) measurements (Fig. S7(a,b)) in a SiO_2(sub)/Ta(1)/Cu(2)/Py(5)/Cu(2)/MgO(1.5)/Ta(1.5) stack, similar to the one studied in the paper but without the MNCN layer. Figure S7(a) exhibits a clear sin2ϕ dependence of the first harmonic voltage, which originates from the planar Hall effect (PHE) as expected. However, the second harmonic voltage (Fig. S7(b)) does not show any significant angular or field dependence associated with SOT unlike the samples with MNCN layer as discussed before. It suggests that the self-induced torque is negligible in our samples for the given thickness of the ferromagnet and other adjacent layers. It is also expected that self-induced torques will be unlikely to produce a significant variation of SOT near the transition temperature of the MNCN. Therefore, the measured SOT can be attributed solely to the MNCN layer.

S4. Computational Details

First principles calculations are performed in three steps. Firstly, the electronic structure is found self-consistently using density functional theory (DFT). Secondly, the Kohn-Sham states are converted to maximally localized Wannier functions (MLWFs). Finally, calculations of the response functions can be performed efficiently within the basis of MLWFs by k-point interpolation.

For the DFT calculation of bulk $Mn_3Ni_{0.35}Cu_{0.65}N$ the code FLEUR[7], a full-potential linearized augmented plane wave method[8], is used. For the exchange correlation functional we chose the Perdew-Burke-Ernzerhof functional within the generalized gradient approximation[9]. The plane-wave cutoff is $11.6\mathrm{a}_0^{-1}$ for the exchange correlation functional and $16.0\mathrm{a}_0^{-1}$ for the charge density, where a_0 is the Bohr radius. The planewave cutoff for the basis functions was $4.0\mathrm{a}_0^{-1}$. For Mn and Ni(Cu) the maximum angular momentum expansion is set to $l_{\max} = 12$ and the muffin-tin radii for each was set to $2.29\mathrm{a}_0$ and for N $l_{\max} = 6$ and the muffin tin radii was $1.29\mathrm{a}_0$. The lattice parameter for the system are $\mathrm{a} = 3.9012$Å from Zhao *et al*[1] as shown in Figure 1(a) of the main text. The $24 \times 24 \times 24$ Monkhorst-Pack k-mesh was sampled in the first Brillouin zone.

From the Bloch wavefunctions obtained from the DFT calculation we construct MLWFs by using the Wannier90 package[10,11] interfaced with the code FLEUR[7]. A mesh of $8 \times 8 \times 8$ k-points was used with 166 Bloch states to obtain 78 MLWFs. Initial projections are chosen to be s, p and d states for Mn and Ni(Cu) and p states for N. The maximum frozen window is set to 4 eV above the Fermi energy. The Hamiltonian and torque operators are evaluated in the Bloch basis and transformed into the MLWF basis.

By Fourier transforming the tight-binding model into k-space and diagonalizing the Hamiltonian, the electric response of the SHE and OHE are evaluated on a dense interpolation k-mesh ($N_{\boldsymbol{k}} = 256 \times 256 \times 256$) using the Kubo formulism,

$$\sigma_{\mathrm{n}\mathbf{k}}^{\mathrm{OH(SH)}} = -\mathrm{e}\hbar \int \frac{d^3k}{(2\pi)^3} \sum_{n\neq m}(f_{\mathrm{n}\mathbf{k}} - f_{\mathrm{m}\mathbf{k}})\mathrm{Im}\left[\frac{\langle u_{n\boldsymbol{k}} | j_y^{O_z} | u_{m\boldsymbol{k}}\rangle\langle u_{m\boldsymbol{k}} | v_x | u_{n\boldsymbol{k}}\rangle}{(E_{n\boldsymbol{k}} - E_{m\boldsymbol{k}} + i\eta)^2}\right] \quad \text{(s9)}$$

where $e > 0$ is the electronic charge, $\hbar$ is the reduced Planck constant, $|u_{m\mathbf{k}}\rangle$ is the periodic part of the Bloch state with crystal momentum $\boldsymbol{k}$, n and m are band indices, $E_{n\boldsymbol{k}}$ is the energy eigenvalue and $f_{n\mathbf{k}}$ is the Fermi-Dirac distribution, v_x is the x component of the velocity operator, η is a smearing term used for numerical convenience, which is set to 25 meV. Finally $j_y^{O_z} = (v_y X_z + X_z v_y)/2$ is the conventional

orbital(spin) current with $X_z = L_z(S_z)$ where L_z is the z component of the orbital angular momentum operator, and S_z is similarly the z component of the spin operator.

S5. The scalar spin chirality imprinted by magnon excitation

We explore the impact of magnon excitations on the scalar spin chirality by numerical modeling. To simplify the calculation, we compute the qualitative behavior of orbital magnetization imprinted by the magnon excitations on a monolayer antiferromagnetic kagome lattice. In our model, only the nearest neighbor Heisenberg exchange interaction is considered, and the Hamiltonian is expressed as:

$$H = J\sum_{i,j} S_i \cdot S_j \qquad \text{(s10)}$$

By taking previous DFT calculations and the Neel temperature of the system into account[3,12], an appropriate value of J is chosen as 6 meV, the length of the spin operator is set as S=3/2, resulting in a Neel temperature of 210K. The magnetization of the system depends on the temperature, and we assume that the average length of the spin moment follows

$$S_T = S(1 - T/T_N)^{\beta}, \text{ with } \beta = 0.362^{13}. \qquad \text{(s11)}$$

Via the linear-spin-wave theory[12,13], the scalar spin chirality (SSC) aroused by magnon excitations is calculated. As shown in Fig. S6(a), an auxiliary small canting angle η=5° is introduced through applying an out-of-plane magnetic field. The calculated SSC as a function of the temperature is shown in Fig. S6(b), and we find that the SSC dependence also has a T^2 dependence at small temperatures. When the system reaches the Néel temperature, no magnons exist in the system, and the SSC magnitude dramatically reduces down to zero. This magnonic generation of SSC leads to a strong orbital response of the spin system, by the mechanism of topological orbital magnetism[12]

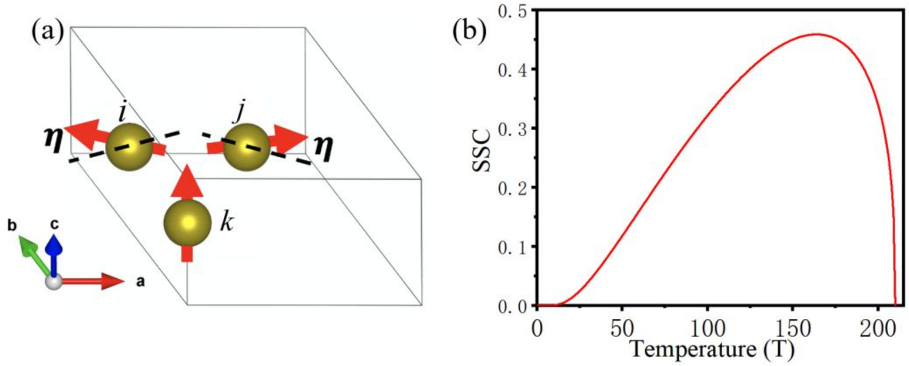

Fig. S8. The scalar spin chirality related to the magnon excitation. (a) Schematic structure of the monolayer antiferromagnetic Kagome lattice under external magnetic field. (b) The value of scalar spin chirality as the function of the temperature.

References

1. Zhao, K. *et al.* Anomalous Hall effect in the noncollinear antiferromagnetic antiperovskite Mn3Ni$_{1-x}$Cu$_x$N. *Phys. Rev. B* **100**, 045109 (2019).

2. Miki, R., Zhao, K., Hajiri, T., Gegenwart, P. & Asano, H. Epitaxial growth and orientation-dependent anomalous Hall effect of noncollinear antiferromagnetic $Mn_3Ni_{0.35}Cu_{0.65}N$ films. *J. Appl. Phys.* **127**, 113907 (2020).

3. Rajan, A. *et al.* Revealing the higher-order spin nature of the Hall effect in non-collinear antiferromagnet $Mn_3Ni_{0.35}Cu_{0.65}N$. *arXiv* **2304.10747**, 1–14 (2023). (accessed 2024-01-01)

4. Manchon, A. *et al.* Current-induced spin-orbit torques in ferromagnetic and antiferromagnetic systems. *Rev. Mod. Phys.* **91**, 035004 (2019).

5. Hayashi, M., Kim, J., Yamanouchi, M. & Ohno, H. Quantitative characterization of the spin-orbit torque using harmonic Hall voltage measurements. *Phys. Rev. B* **89**, 144425 (2014).

6. Roschewsky, N. *et al.* Spin-orbit torque and Nernst effect in Bi-Sb/Co heterostructures. *Phys. Rev. B* **99**, 195103 (2019).

7. Wortmann, D. ; Michalicek, G. ; Baadji, N. ; Betzinger, M. ; Bihlmayer, G. ; Bröder, J. ; Burnus, T. ; Enkovaara, J. ; Freimuth, F. ; Friedrich, C. ; Gerhorst, C.-R. ; Granberg Cauchi, S. ; Grytsiuk, U. ; Hanke, A. ; Hanke, J.-P. ; Heide, M. ; H, S. FLUER. Relaxed thin film structures of one, two, and three magnetic 3d transition metal layers on FCC noble-metal substrates based on FLAPW PBE calculations. *Materials Cloud Archive **2023.180*** (2023) https://doi.org/10.24435/materialscloud:dk-wq.

8. Wimmer, E., Krakauer, H., Weinert, M. & Freeman, A. J. Full-potential self-consistent linearized-augmented-plane-wave method for calculating the electronic structure of molecules and surfaces: O2 molecule. *Phys. Rev. B* **24**, 864–875 (1981).

9. Perdew, J. P., Burke, K. & Ernzerhof, M. Generalized Gradient Approximation Made Simple. *Phys. Rev. Lett.* **77**, 3865–3868 (1996).

10. Wang, X., Yates, J. R., Souza, I. & Vanderbilt, D. Ab initio calculation of the anomalous Hall conductivity by Wannier interpolation. *Phys. Rev. B* **74**, 195118 (2006).

11. Pizzi, G. *et al.* Wannier90 as a community code: new features and applications. *J. Phys. Condens. Matter* **32**, 165902 (2020).

12. Zhang, L.-C. *et al*. Imprinting and driving electronic orbital magnetism using magnons. *Commun. Phys* **3**, 227 (2020).

13. Mook, A. *et al.* Magnon Hall effect and topology in kagome lattices: A theoretical investigation. *Phys. Rev. B* **89**, 134409 (2014).
